# Resistance to *Elsinoë Ampelina* and Expression of Related Resistant Genes in *Vitis Rotundifolia* Michx. Grapes

**DOI:** 10.3390/ijms12063473

**Published:** 2011-06-01

**Authors:** Clifford Louime, Jiang Lu, Oghenekome Onokpise, Hemanth K. N. Vasanthaiah, Devaiah Kambiranda, Sheikh M. Basha, Hae Keun Yun

**Affiliations:** 1 College of Engineering Sciences, Technology and Agriculture, Florida A&M University, Tallahassee, FL 32317, USA; E-Mails: jiang.lu@hotmail.com (J.L.); o.onokpise@att.net (O.O.); hemanth.vasanthaiah@gmail.com (H.K.N.V.); devaiah.kambiranda@gmail.com (D.K.); sheikh.mehboob@yahoo.com (S.M.B.); 2 National Horticultural Research Institute, Fruit research Division, Rural Development Administration, 475 Imok-Dong, Jangan-Gu, Suwon 440-706, Korea; E-Mail: haekeun.yun@yahoo.com

**Keywords:** anthracnose disease resistance, Elsinoë ampelina, pathogenicity testing, Vitis rotundifolia

## Abstract

Muscadine grapes (*Vitis rotundifolia* Michx) are considered as excellent genetic resources for grape breeding programs as they are known for their hardiness and resistance to pests and diseases. However, contrary to popular belief, our study indicated that not all muscadine cultivars are resistant to anthracnose disease. In order to identify a source of genetic tolerance towards anthracnose among muscadine cultivars, a series of *in-situ* and *ex-situ* experiments were conducted through strict and sensitive screening processes. Two consecutive years of field evaluation of 54 grape cultivars showed various levels of anthracnose incidence among the cultivars between a scale of 0 (tolerant) to 5 (highly-susceptible). Resistance bioassay by inoculation of different spore densities of *Elsinoë ampelina* on 40 cultivars presented similar results and was consistent with those obtained from the field test. A real-time PCR analysis was conducted to investigate differences of gene expression between susceptible and tolerant cultivars and to confirm results by phenotypic identification. Expression of genes encoding chalcone synthase, stilbene synthase, polygalacturonase-inhibiting protein, chitinase and lipid transfer-protein was only detected in tolerant cultivars. Resistant muscadine cultivars identified in this study could be excellent candidates for grape disease resistance breeding programs.

## 1. Introduction

Anthracnose of grapes is an economically devastating disease caused by the fungus *Elsinoë ampelina* Shear. Symptoms usually appear as numerous circular spots, which enlarge then become sunken and produce lesions with round edges. Once established in a vineyard, the disease can be very destructive. The pathogenic fungus, which attacks all aerial parts of the plants, such as fruits, leaves, tendrils and petioles, is of considerable economic importance [1–3]. The fungus over-winters in dormant and dead canes, making it very difficult to control. Strategies for the control of anthracnose in grapevines, such as developing resistant cultivars are necessary in order to reduce the production cost and environmental impacts of fungicide applications in areas of high disease pressure. For this purpose, the selection of genetic resources showing tolerance to anthracnose is a prerequisite for any breeding program.

It has been reported that, among the grape species, *Vitis vinifera* is highly susceptible, whereas *Vitis labrusca* and *Vitis sp.* (hybrids) are resistant or moderately resistant [1,4,5], while *Muscadinia rotundifolia* Milch. is immune to *E. ampelina* [6,7]. *Vitis vinifera* is one of the finest grapes grown in the world both for table and wine purposes. On the other hand, native muscadine grapes have been considered as one of the most valuable genetic resources in breeding programs for grape disease tolerance [5,6,8–13]. As anthracnose is highly prevalent in this part of the world, it is one of the principal factors preventing the development of a grape industry using *V. vinifera* in the southeastern United States [3]. Growers in this area are forced to grow local species, such as muscadine and Florida hybrid bunch grapes that often compromise the fruit quality. Muscadine grapes have been known for their tolerance or ‘tolerance’ to many diseases found in bunch (*Euvitis* Planch.) grape species [6,7].

Evaluating and screening of perennial crops, including grapes, for disease tolerance is a constant challenge. Several native grapes and other cultivars (*Vitis sp.*) have been evaluated for their tolerance to anthracnose [4–7]. This process or approach is time-consuming, laborious and costly. Recently, Yun *et al.* [12] have developed an efficient and reliable screening process for selecting grape cultivars resistant to anthracnose based on pathogen inoculation and by the application of culture filtrates from *E. ampelina*, which is accurate, economical and labor-saving.

As of yet, there have been only two formal reports of anthracnose or its causal agent in muscadine grapes [13,14]. Pierce’s disease has prevented growing *V. vinifera* in Floridian and Southeastern United States regions. Muscadine and Florida hybrid bunch grapes can be successfully grown as they are tolerant to Pierce’s disease, but their tolerance level to anthracnose varies. The breeding work at the Center for Viticulture and Small Fruit Research, Florida A&M University, Tallahassee, Florida, USA has been hampered due to lack of knowledge about the anthracnose tolerance levels in muscadine cultivars used in the breeding program. In a study in 2006 and 2007, 21 (40%) of the 51 muscadine cultivars maintained in the vineyard showed anthracnose symptoms, which were found mainly on young leaves and tendrils as circular or irregular black spots. Hence it was necessary to investigate the level of tolerance of the muscadine cultivars at hand, and to use a more stringent screening process to select muscadine genetic resources that are resistant to anthracnose for use in the ongoing breeding process.

The objectives of this study were therefore to identify the pathogen isolated from the muscadine grapevines and to evaluate the disease tolerance potential of muscadine grape genotypes by a combination of screening approaches. These included disease scoring after vineyard inspection, susceptibility testing via bioassay culture filtrates, and molecular biology techniques such as gene [chalcone synthase (*CHS*), stilbene synthase (*StSy*), polygalacturonase inhibiting protein (*PGIP*), chitinase (*CHI*) and lipid transfer protein (*LTP*)] expression studies following fungal inoculation. These genes are known to be involved in fungal disease development of fruit crops. Findings from these studies would help identify muscadine cultivars truly tolerant to anthracnose to incorporating into our breeding programs.

## 2. Results and Discussion

### 2.1. Pathogen Isolation and Characterization

**Figure 1:**
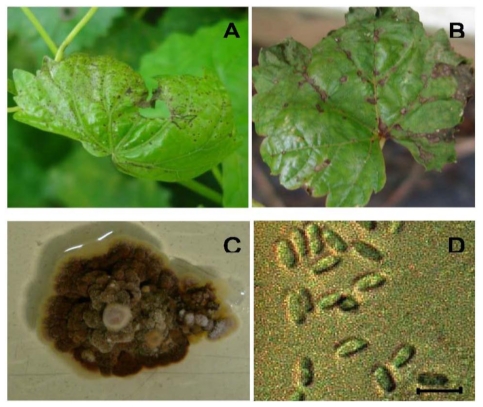
Anthracnose symptoms on the grapevine leaves and the pathogen (*Elsinoe*) isolated from the infected leaves. **(A)** Naturally infected leaf in the vineyard; **(B)** artificially infected leaf with pathogen spore suspension; **(C)** *Elsinoe* colony on PDA; and **(D)** *Elsinoe* spores under the microscope (×400). Bar represents 20 μm.

**Figure 2:**
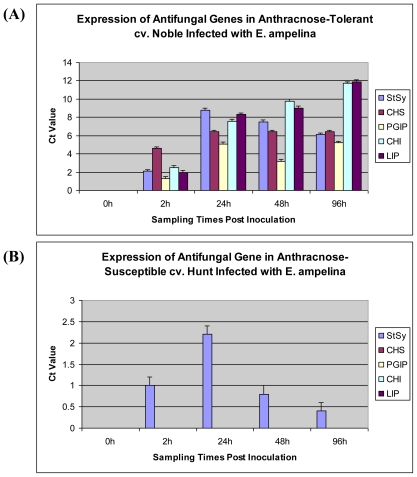
Expression of antifungal genes based on *C*_t_ values (average of three reactions) measured through real-time PCR at different time periods post inoculation in muscadine cultivar **(A)** Noble (anthracnose-tolerant) and **(B)** Hunt (anthracnose-susceptible). Ubiquitin was used as an internal control in this experiment—*StSy* (Stilbene Synthase)—*CHS* (Chalcone Synthase)—*PGIP* (Polygalacturonase Inhibiting Protein)—*CHI* (Chitinase)—*LIP* (Lipid Transfer Protein).

Fungus isolated from infected muscadine grapevine leaves showed slow growth (3.5 cm in diameter in 10 days) and dark red mounds with some mycelia on PDA (Figure 1C). Spores ranged from 11.0 to 16.5 μm × 3.9 to 5.7 μm, cylindrical and hyaline with pointed ends (Figure 1D), which was consistent with previous reports for *E. ampelina* (3). The fungus was identified not only by microscopic observations of morphological characteristics, but also by PCR amplification of fungal DNA. Electrophoresis of the obtained PCR products on agarose gel (1.2%) showed a single expected 500 bp amplified band (Figure 2). These results clearly confirmed that the fungus isolated from the lesions of the muscadine grapevine leaves (cv. ‘Hunt’) was the same species as *E. ampelina*, the causal agent of anthracnose in grapevines.

### 2.2. Pathogenicity Testing

To test for possible pathogenicity of the fungus, the incidence of symptoms was investigated on anthracnose-tolerant and susceptible cultivars after spraying with fungal spore suspension (2 × 10^5^ spores per mL). Necrotic lesions associated with the anthracnose fungus appeared 4 days after inoculation with fungal spore suspension on cvs. ‘Cabernet Sauvignon’ (*V. vinifera*) and ‘Hunt’ (*M. rotundifolia*) (Figure 1B). The fungus isolated from anthracnose lesions of muscadine grapevine leaves had high pathogenicity (was virulent) and produced anthracnose disease symptoms on cultivars ‘Cabernet Sauvignon’ and ‘Hunt’. Further inoculation using the above isolated fungus to study the transcriptome analysis of the anti-fungal genes in both *Elsinoë -*tolerant and -susceptible cultivars substantiates that the leaf samples used were infected with *E. ampelina*.

### 2.3. In-Situ and Ex-Situ Analysis to Determine the Tolerance Level of Muscadine Cultivars

Both *in-situ* and *ex-situ* analysis were carried out to screen the grape cultivars tolerant to anthracnose for further use in our breeding program. The incidence of anthracnose symptoms was rated based on their natural infection in the vineyard and the varietal responses were evaluated through bioassay using culture filtrates from fungus *Elsinoë*. Finally, the tolerance level of muscadine cultivars was tested using gene expression studies of selected defense-related genes.

### 2.4. Anthracnose Incidence in the Field

Vineyard investigation in 2006 and 2007 pointed out that the anthracnose symptoms were visible on the leaves, tendrils and stems of the muscadine grapevines. Prior to 2006, the incidence of anthracnose on muscadine cultivars was not significant. The level of shoot infection varied among the *M. rotundifolia* genotypes was considered to be immune to anthracnose (Table 1). Among the 54 muscadine cultivars studied 23 cultivars were found to be immune to *Elsinoë* infestation, 12 cultivars showed incidence ≤1, 16 cultivars showed incidence between 1.1 to 4.2 on a 0 to 5 scale. Among the muscadine grape cultivars tested, cultivars ‘Janet’, ‘Scarlet’, ‘Digby’, and ‘Watergate’ had the highest incidence score of 4.2, 3.1, 2.5 and 2.5, respectively. This data clearly shows that the muscadine cultivars are not immune to anthracnose infection. In the case of the anthracnose susceptible *Vitis* sp. (cv. Blanc du Bois and Orlando Seedless) and *V. vinifera* (cv. Cabernet Sauvignon), the shoot incidence was recorded 5 with maximum incidence revealing their venerability to anthracnose.

### 2.5. Bioassay with Culture Filtrates

Further validation of the 36 muscadine grapevine and 4 *Vitis sp.* cultivars to anthracnose tolerance was carried out employing Yun *et al.*’s [12] screening system using culture filtrates from *E. ampelina*. The results of the bioassay with culture filtrates showed that some cultivars were tolerant, some were susceptible and while others were moderately resistant. All of the cultivars except ‘Late Fry’, ‘Noble’, ‘Pam’, ‘Senoi’, ‘Southern Home’, ‘Sweet Jenny’ and ‘Welder’ developed necrosis after treatment with *Elsinoë* culture filtrate (1:1 dilution) on the wounded surface (Table 2). Eleven cultivars developed necrosis incidence at 1:4 dilution of the culture filtrate and 4 of them at 1:8 dilution. The development of necrosis in the anthracnose-susceptible cultivars (*Vitis sp.* cv. ‘Blanc du Bois’ and ‘OrlandoSeedless’; *V. vinifera* cv. ‘Chardonnay’ and ‘Cabernet Sauvignon’) was significantly higher than in the tolerant cultivars. The leaf of Florida hybrid bunch grape cv. ‘Blanc du Bois’ developed necrosis of 2–3 mm over the wounded spot, even at 1:16 dilution. This study also clearly demonstrates that not all muscadine cultivars are immune to anthracnose disease. The spectrum of sensitivity to the culture filtrates was highly consistent with susceptibility to anthracnose in a number of grapevine cultivars observed during the vineyard investigation.

### 2.6. Gene Expression Studies during the Course of Infection

In order to further validate the anthracnose tolerance level of different grape cultivars, real-time PCR analysis was carried out with the selected defense-related genes. Based on the field investigation and bioassay analysis, five anthracnose-tolerant and -susceptible cultivars along with one Florida hybrid bunch and *V. vinifera* cultivars were randomly selected for this study. The choice of the primers was based on ESTs, genes and mRNA sequences of *Vitis sp.* found in the public domain. Initial standard PCR amplification revealed that the primer pairs targeted a single gene within a given gene family, indicating good quality and absence of genomic contamination in the template cDNA (Data not shown). Ubiquitin was used as the internal control. The genes encoding chalcone synthase (*CHS*), stilbene synthase (*StSy*), polygalacturonase-inhibiting protein (*PGIP*s), chitinase (*CHI*) and lipid-transfer protein (*LIP*) were highly expressed in anthracnose-tolerant cultivar ‘Noble’ (*M. rotundifolia*) upon *Elsinoë* inoculation (Figure 2) but were completely absent in susceptible cv. ‘Hunt’ (*M. rotundifolia*), ‘Blanc du Bois’ (*Vitis sp.*) and ‘Cabernet Sauvignon’ (*V. vinifera*) based on the *C*_t_ values obtained through real-time PCR analysis. Except for *StSy* expression, which was observed at low levels in anthracnose-susceptible cultivar ‘Hunt’.

A similar pattern of expression was also observed in the other anthracnose-tolerant muscadine cultivars studied viz., ‘Pam’, ‘Senoi’, ‘Southern Home’ and ‘Welder’ and anthracnose-susceptible muscadine cultivars studied viz., ‘Fry Seedless’, ‘Granny Val’, ‘Higgins’ and ‘Janet’ (data not shown), further confirming their tolerance level. This further indicates that variability exists among muscadine and other grape cultivars for anthracnose tolerance. The expression levels of all the five genes studied in muscadine varied during the course of *Elsinoë* infection, indicating their role in anthracnose tolerance.

### 2.7. Discussion

The level of tolerance to *E. ampelina* varies among different cultivars of grapes that affect their production [12,19]. Until now it was believed that most of the muscadine grapevines are immune to anthracnose caused by *E. ampelina*. However, we recently observed the incidence of anthracnose on 40% of the leaves, tendrils and stems of muscadine cultivars grown at our Center. This observation led us to this study to analyze anthracnose tolerance among muscadine cultivars through strict and stringent process.

Two consecutive years of field study showed variation in the level of shoot infection among the muscadine cultivars to *E. ampelina* infection (Table 1). The incidence of disease varied among muscadine cultivars from ≤1 to 4.2 on a 0 to 5 scale, where as anthracnose susceptible *Vitis sp.* cultivars studied documented 5.0, disclosing their susceptible to anthracnose. Among the muscadine cultivars studied 45% of them were found immune to anthracnose, while cultivars ‘Janet’, ‘Scarlet’, ‘Digby’, and ‘Watergate’ were found highly susceptible. The above study revealed that all muscadine grape cultivars were not immune or highly resistant to anthracnose disease. Further, the tolerance to anthracnose in various muscadine grape cultivars was evaluated by using bioassay with culture filtrates from the pathogen. These results were consistent with those from field tests. Susceptible cultivars were found sensitive to eight-fold diluted culture filtrates, but resistant cultivars were not affected, even by the original culture filtrates. A similar pattern has been reported in apple with AM-toxins from *Alternaris mali* [20,21] and pear leaves with AK-toxins from *A. kikuchiana* [22,23].

**Figure 3:**
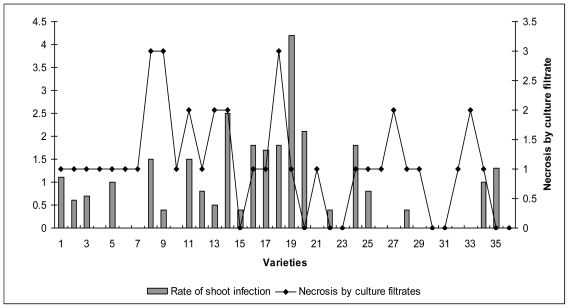
Comparison between anthracnose incidence in vineyard and necrosis resulted from bioassay of culture filtrates from *Elsinoe ampelina*. 1; ‘African Queen’, 2; ‘Alachua’, 3; ‘Albermale’, 4; ‘Black Beauty’, 5; ‘Carlos’, 6; ‘Cowart’, 7; ‘Darlene’, 8; ‘Dixie Land’, 9; ‘Dixie Red’, 10; ‘Early Fry’, 11; ‘Farrer’, 12; ‘Florida Fry’, 13; ‘Fry’, 14; ‘Fry Seedless’, 15; ‘Golden Isle’, 16; ‘Granny Val’, 17; ‘Higgins’, 18; ‘Hunt’, 19; ‘Janet’, 20; ‘Late Fry’, 21; ‘Loomis’, 22; ‘Noble’, 23; ‘Pam’, 24; ‘Pineapple’, 25; ‘Pride’, 26; ‘Regale’, 27; ‘Rosa’, 28; ‘Scarlet’, 29; ‘Scupernong’, 30; ‘Senoia’, 31; ‘Southern Home’, 32; ‘Southern Land’, 33; ‘Sugargate’, 34; ‘Sugarpop’, 35; ‘Sweet Jenny’, 36; ‘Welder’. 
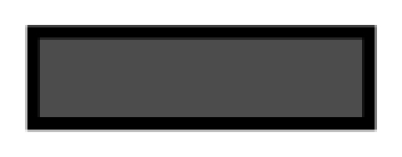
; Rate of shoot infection in vineyard, 
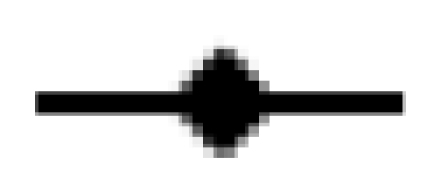
; Necrosis by bioassay of culture filtrates from *E. ampelina*. Score range as described above in Table 1 and Table 2.

A comparative analysis of both field and bioassay studies revealed that 19 muscadine cultivars (‘Black Beauty’, ‘Carlos’, ‘Cowart’, ‘Darlene’, ‘Early Fry’, ‘Florida Fry’, ‘Golden Isle’, ‘Loomis’, ‘Pride’, ‘Noble’, ‘Pam’, ‘Regale’, ‘Scarlet’, ‘Scupernong’, ‘Senoi’, ‘Southern Home’, ‘Southern Land’, ‘Sugar Pop’ and ‘Welder’) among 51 studied were immune to anthracnose infection (Figure 3) showing 0 to ≤1 scale shoot infection and 0 to 1 (slight necrosis) after *Elsinoë* infection. Appearance of slight necrosis on the leaf of few cultivars may be due to tissue damage during artificial inoculation and infection, which is not significant. This study also clearly demonstrates that not all muscadine cultivars are immune to anthracnose disease. The spectrum of sensitivity to the culture filtrates was highly consistent with susceptibility to anthracnose in a number of grapevine cultivars observed during the vineyard investigation. The muscadine cultivars with 0 to 1.5 scale showing necrotic lesions covering up to 10% of leaf area can be successfully considered for grape breeding program. The infection of *Elsinoë* and appearance of slight necrosis on leaf of few cultivars may be attributed to a hypersensitive reaction.

Further validation of anthracnose tolerance level of different grape cultivars was carried out using real-time PCR analysis and selective antifungal specific genes. Expression of Chalcone synthase (*CHS*), stilbene synthase (*StSy*), polygalacturonase-inhibiting protein (*PGIP*s), chitinase (*CHI*) and lipid-transfer protein (*LIP*) were found only in the anthracnose-tolerant muscadine cultivars studied (Figure 2A). Expression of these genes was rapid 24 h of *Elsinoë* inoculation. Similar validation of expression of pathogenesis-related genes has been recorded in grapevine against *Uncinula necator* that causes powdery mildew and rupestris stem pitting-associated virus (*GRSPaV*) [24,25]. Chalcone synthase is a phytoalexin biosynthetic enzyme [26], which is involved in defense against fungal diseases. CHS catalyses a key step in the synthesis of many secondary compounds with demonstrated antifungal activity [27] in *Arabidopsis* against the growth of *Pythium mastophorum*, which causes root rot [28]. Higher expression of *CHS* in anthracnose-tolerant muscadine cv. ‘Noble’ and other cultivars studied clearly indicates its tolerance mechanism.

## 3. Experimental Section

### 3.1. Plant Materials

The field-grown muscadine and Florida hybrid bunch grape cultivars maintained at the vineyard of the Center for Viticulture and Small Fruit Research, Tallahassee, Florida were used for field and bioassay studies. Based on the results obtained for further transcriptome analysis, two-year-old greenhouse-grown *M. rotundifolia* (cvs. ‘Noble’, ‘Pam’, ‘Senoi’, ‘Southern Home’ and ‘Welder’ (anthracnose-tolerant); ‘Fry Seedless’, ‘Granny Val’, ‘Higgins’, ‘Hunt’ and ‘Janet’ (anthracnose-susceptible)), *Vitis vinifera* (cv. ‘Cabernet Sauvignon’) and *Vitis sp.* (cv. Blanc du Bois) were used after challenging with *E. ampelina*. The greenhouse-grown plants were derived from the cuttings made from the greenhouse-maintained grape genotypes at Center for Viticulture and these plants were grown in three-gallon pots.

### 3.2. Isolation and Characterization of the Fungus

To identify *Elsinoë ampelina*, anthracnose infected leaves of muscadine grapevine cv. Hunt were collected from the experimental vineyard at the Center for Viticulture and Small Fruit Research in Tallahassee, Florida (Figure 1A). The leaf surface was disinfected by dipping in 2% sodium hypochlorite solution for 1 min followed by 75% ethyl alcohol and then rinsed in distilled water. The leaves were placed on potato dextrose agar (PDA) medium and incubated at 28 °C under a fluorescent light. The fungus developed as a dark red mound and was isolated on PDA medium as single-conidia cultures. Single colony cultures were transferred to new plates. Colony type and spore appearance of this fungus were investigated by microscopic observations and compared with previous reports for *E. ampelina*. DNA isolated from the fungus was also analyzed by PCR amplification using the following 18sRNA based primers, 5′-TCCGTAGGTGAACCTGCGGA-3′ (left) and 5′-TCCTACCTGATCCGAGGTCA-3′ (right), designed based upon alignment of *E. ampelina* genes deposited in the NCBI database. Genomic DNA obtained from the isolate grown on Fries liquid medium [15] was amplified following the protocol of Müller *et al.* [16].

### 3.3. Culture and Spore Production

Several colonies of the pathogenic fungus were transferred to Fries liquid medium and incubated in a shaker incubator (140 rpm) at 28 °C for 10 days. Fungal cultures harvested by centrifugation were suspended in sterile distilled water by homogenization, poured on V-8 juice agar medium and incubated for 2 days at 28 °C under a near ultraviolet lamp for spore production. To harvest pathogenic spores, sterile distilled water was used to scrape colonies off the plates. The harvested spores were adjusted to different concentrations with sterile distilled water, and then used to inoculate the grapevine leaves.

### 3.4. Pathogenicity Test

Greenhouse-grown plants were used for this study. Spore suspension adjusted to 2 × 10^5^ conidia per mL was sprayed onto *V. vinifera* (cv. ‘Cabernet Sauvignon’) and *M. rotundifolia* (cv. ‘Hunt’) grape cultivars, whereas control plants were sprayed with distilled water. Treated plants were immediately incubated in a humid chamber (28 °C) for 48 h, and moved to the greenhouse. The plants were inspected for appearance of symptoms on the leaves, and the degree of symptom development was recorded.

### 3.5. Disease Scoring

Field test data were collected from the experimental vineyard at the Center for Viticulture and Small Fruit Research, Florida A&M University in Tallahassee, Florida. The field-grown plants were investigated for incidence of lesions due to anthracnose during the spring, summer and autumn of 2006 and 2007. Disease severity was assessed by counting the number of lesions and rating the symptom expression on a scale of 0 (no necrosis) to 5 (severely infected). The incidence of anthracnose was recorded from the lesions of 10 leaves on the upper part of the shoots from the shoot tip and on the shoots of 54 cultivars. The data was collected for three replicates (Table 1).

### 3.6. Bioassay with Culture Filtrates

After incubating the pathogen in Fries medium at 28 °C for 10 days, cell-free culture filtrates (*CFCF*) of *E. ampelina* were collected from the supernatant by centrifugation at 10,000 × g for 5 min using a table top centrifuge (Eppendorf, Centrifuge 5415C) and sterilized by ultrafiltration (0.2 μm pore diameter). Muscadine grapevine leaves from 36 different cultivars located at the experimental vineyard in Tallahassee, FL were used for this study. Five different leaves from either upper third or fourth leaf from the shoot apex were collected from four different grape plants of each cultivar and brought to the lab on ice. These leaves were surface sterilized with 75% ethanol, dipped in 2% sodium hypochlorite for 15 s and rinsed in distilled water. Later these leaves were injured with a needle tip and 30 μL of culture filtrate, diluted to 1:1, 1:4, 1:8 and 1:16 (v/v) with distilled water was deposited onto the wounded portion of the leaves. Fresh Fries medium was applied to the wounded portions of grapevine leaves as control. Leaves treated with culture filtrates and the control medium were incubated in a dark, moist chamber (>95% RH) for 3 days at 28 °C. The area of the necrotic lesion around the wound was measured to evaluate the tolerance of different cultivars.

### 3.7. Gene Expression Studies

#### 3.7.1. Pathogen Inoculation

Plants grown under controlled greenhouse conditions were used in this study. For inoculation studies, spore suspension adjusted to 2 × 10^5^ conidia per mL were sprayed onto young *M. rotundifolia* cv. ‘Noble’ ‘Pam’, ‘Senoi’, ‘Southern Home’ and ‘Welder’ (anthracnose-tolerant); ‘Hunt’, ‘Digby’, ‘Janet’, ‘Scarlet’ and ‘Watergate’ (anthracnose-susceptible)), *V. vinifera* (cv. ‘Cabernet Sauvignon’) and *Vitis sp.* (cv. ‘Blanc du Bois) plants as treatment and three plants of each cultivar were sprayed with distilled water as the control. The tolerance and susceptible of muscadine cultivars to anthracnose was based on field observance. For optimization of lesion formation, inoculated plants were incubated in a humid chamber (28 °C) for 48 h, and later moved to the greenhouse. Leaf samples were randomly collected at 0 (before inoculation), 2, 24, 48 and 96 h for RNA isolation. Sample collection was stopped after four days post inoculation as the lesions appeared on leaves and young shoots at this time. Anthracnose susceptible and tolerant muscadine cultivars were randomly selected based on the results of field and bioassay analysis.

#### 3.7.2. RNA Extraction and Analysis

Total RNA from uninfected and *Elsinoë* infected leaf tissue was isolated using modified guanidine thiocyanate extraction method [17]. The yield and quality of total RNA products were measured by absorbance at 230, 260, and 280 nm (A_260/230_ and A_260/280_ ratios) using a Nano Drop spectrophotometer (Nano Drop, Technologies Inc.) and by electrophoresis on a 1.5% non-denaturing agarose gel [18].

#### 3.7.3. Primer Design

For further validation, primers specific to *Vitis* species defense-related genes were designed to check the expression levels of these genes upon challenging with *Elsinoë* in both tolerant and susceptible grapevine cultivars. Oligonucleotide primers for chalcone synthase (*CHS*), stilbene synthase (*StSy*), polygalacturonase inhibiting protein (*PGIP*), chitinase (*CHI*) and lipid transfer protein (*LTP*) were designed based on sequences conserved among different *Vitis* species including *V. vinifera*, *V. labrusca*, *V. shuttleworthii*, *V. riparia*, and *M. rotundifolia*. The primers for qPCR were designed using Primer3 program (http://primer3.sourceforge.net/). Primers were synthesized by WGC (Saint Louis, MO). Primer sequences are provided in Table 3.

#### 3.7.4. Quantitative PCR

Real-time PCR was performed to confirm the expression of defense-related genes in anthracnose-tolerant and -susceptible grapevine cultivars. Total RNA was treated with DNase (QIAGEN) to remove DNA pollution and subsequently purified with the RNeasy Cleanup Kit (QIAGEN). RNA was reverse transcribed to cDNA by means of the iScript reverse transcription system (Bio-Rad). Prior to real-time PCR analysis, a standard control PCR was carried out to check for the presence of genomic DNA contamination. PCR reactions contained 2 μL of diluted cDNA, 2 μL of 10 × PCR Buffer (Promega), 0.4 μL 10 mM dNTPs (Promega), 1.6 μL 2 mM MgCl_2_, 1 U *Taq* DNA polymerase (Promega), and 2 μL of each gene-specific primer (10 μM/μL, Operon Biotechnologies, Inc) and were brought to a final reaction volume of 20 μL with PCR-grade water. Reactions were incubated at 95 °C for 2 min initial denaturation and then cycled at 95 °C for 30 s, 60 °C for 30 s (for all genes), and 72 °C for 1 min for a total of 35 cycles followed by a final extension of 5 min at 72 °C.

Quantitative or real time PCR was performed on IQ5Cycler (Bio-Rad) using a SYBR-green mix from the manufacturer. Reactions were performed in 20 μL including 20 ng RNA, 0.3 μM of each primer and 5 μL SYBER-green mix. All reactions were performed in triplicate to ensure reproducibility of the results. Amplification was carried out with one cycle at 95 °C for 15 min, eight cycles at 94 °C for 30 s, 60 °C for 40 s and, 30 cycles at 94 °C for 10 s and 60 °C for 30 s. Melting curves of the amplified products were recorded. Relative mRNA level for each sample was calculated using the relative *C*_t_ method (level = 2 (C_t_ of the no RT control − C_t_ of the sample)), with *C*_t_ being the cycle number at which fluorescence surpassed background (determined during the first 10 cycles of amplification). Results were analyzed using the ICycler system sequence detection software V1.3 (Bio-Rad). Data were normalized against expression of the housekeeping gene ubiquitin.

## 4. Conclusions

Plants respond to pathogen infestation by expressing genes encoding defense-related proteins which are believed to play a role in plant defense. Polygalacturonase-inhibiting proteins are extracellular plant proteins capable of inhibiting fungal endopolygalacturonases (*PG*s) [29] thereby protecting the plants against pathogens. Whereas chitinases (*CHI*) are capable of hydrolyzing chitin-containing fungal cell walls and therefore play a major role in plant defense [30]. Lipid-transfer protein (*LTP*) has been reported to exhibit antifungal activity and is also involved in triggering many important cell-signaling and metabolic pathways upon fungal infection [31].

Stilbene synthase expression was also found at higher levels in anthracnose-tolerant cv. ‘Noble’ and other cultivars studied. However, expression of stilbene synthase genes was also found at lower levels in susceptible cultivars (Figure 2b), because their production has been reported to occur even in the absence of the usual stimulus [32,33]. Phytoalexins are considered as strong fungistatic substances [34]. The role of phytoalexins in defense has been demonstrated in several crops [35,36], including the grapevine [37]. Stilbene synthase produces *trans-*resveratrol, the major phytoalexin in the plant. This triphenol is subsequently metabolized into other phytoalexins of grapevine. Resveratrol plays an important role in tolerance to colonization by fungi and exhibits outstanding biological properties in human health [37]. The transgenic plant possessing grapevine stilbene synthase genes are known to improve plant tolerance to fungal diseases particularly to downy mildew (*Pseudoperonospora humuli*), powdery mildew (*Podosphaera macularis*), *Botrytis cinerea*, *Eutypa lata*, *Plasmopara viticola* and *Phomopsis viticola* [38–41]. The expression of stilbene synthase gene in anthracnose-tolerant cv. ‘Noble’ and other tolerant muscadine cultivars indicates its possible tolerance mechanism within the plant against the fungal pathogen *E. ampelina*. This clearly shows that the induction of these antifungal genes in anthracnose-tolerant muscadine cultivars may indeed reduce the damage caused by *Elsinoë*. Anthracnose tolerance level varies widely among muscadine genotypes and the tolerant genotypes produce several defense-related genes to overcome pathogen infection. Through accurate screening of muscadine grape germplasm for anthracnose disease tolerance by bioassay with specific toxic compound produced from pathogen, pathogen inoculation, and field tests coupled with gene expression studies, it is possible to select resistant muscadine grape genetic resources to be utilized in breeding programs. This study also demonstrates an efficient screening system could be a valuable tool in examining the degree of tolerance in muscadine grape cultivars for future use in grape crop improvement.

## Figures and Tables

**Table 1:** Incidence of anthracnose in muscadine grapevines in the vineyards.

Variety	Shoot infection	Variety	Shoot infection
African Queen	1.1 ± 0.17	Loomis	0
Alachua	0.6 ± 0.11	Magnolia	0.8 ± 0.17
Albermale	0.7 ± 0.17	Nesbitt	0
Black Beauty	0	Noble	0.4 ± 0.15
Black Fry	0	Pam	0
Carlos	1.0 ± 0.33	Pineapple	1.8 ± 0.24
Cowart	0	Pride	0.8 ± 0.17
Darlene	0	Regale	0
Digby	2.5 ± 0.33	Rosa	0
Dixie	0	Scarlett	3.1 ± 0.48
Dixie Land	1.5 ± 0.18	Scupernong	0.4 ± 0.11
Dixie Red	0.4 ± 0.11	Senoia	0
Doreen	0	Southern Home	0
Early Fry	0	Southern Land	0
Farrer	1.5 ± 0.23	Sterling	0
Florida Fry	0.8 ± 0.15	Sugargate	0
Fry	0.5 ± 0.15	Sugarpop	1.0 ± 0.15
Fry Seedless	2.5 ± 0.56	Summit	1.3 ± 24
Golden Isle	0.4 ± 0.11	Supreme	0
Granny Val	1.8 ± 0.43	Sweet Jenny	1.3 ± 0.18
Higgins	1.7 ± 0.37	Tara	0
Hunt	1.8 ± 0.24	Triumph	0
Ison	0	Watergate	2.5 ± 0.17
Janebell	0	Welder	0
Janet	4.2 ± 0.58	Blanc du Bois	5.0 ± 0.24
Jumbo	2.1 ± 0.29	Orlando Seedless	5.0 ± 0.29
Late Fry	2.1 ± 0.24	Cabernet Sauvignon	5.0 ± 0.24

* Incidence of anthracnose was expressed as mean number (±SE, n = 9) of shoots with lesions from 10 leaves in upper part of shoots from the shoot tip, and on the shoots in the vineyard. Score range from 0 (tolerant) to 5 (highly susceptible), where 0 = no necrosis; 1 = necrotic lesions covering 10% of the leaf area; 2 = necrotic lesions covering 20% of the leaf area; 3 = necrotic lesions covering 50% of the leaf area; 4 = necrotic lesions covering 75% of the leaf area; and 5 = necrotic lesions covering 90% of the leaf area.

**Table 2:** Comparison of different grape cultivars in their responses to the culture filtrates of *E. ampelina*.

Variety	Dilution of culture filtrates				Variety	Dilution ofculture filtrates			
	1:1	1:4	1:8	1:16		1:1	1:4	1:8	1:16
African Queen	1^z^	0	0	0	Loomis	1	0	0	0
Alachua	1	0	0	0	Noble	0	0	0	0
Albermale	1	0	0	0	Pam	0	0	0	0
Black Beauty	1	0	0	0	Pineapple	1	0	0	0
Carlos	1	0	0	0	Pride	1	0	0	0
Cowart	1	0	0	0	Regale	1	0	0	0
Darlene	1	1	1	0	Rosa	2	1	0	0
Dixie Land	3	2	0	0	Scarlett	2	1	0	0
Dixie Red	3	2	1	0	Scupernong	1	0	0	0
Early Fry	1	1	0	0	Senoia	0	0	0	0
Farrer	2	1	0	0	Southern Home	0	0	0	0
Florida Fry	1	0	0	0	Southern Land	1	0	0	0
Fry	2	2	1	0	Sugargate	2	1	0	0
Fry Seedless	2	1	1	0	Sugarpop	1	0	0	0
Golden Isle	0	0	0	0	Sweet Jenny	0	0	0	0
Granny Val	1	0	0	0	Welder	0	0	0	0
Higgins	1	0	0	0	Blanc du Bois	4	3	3	3
Hunt	3	2	1	0	Orlando Seedless	4	2	2	0
Janet	1	0	0	0	Chardonnay	3	2	0	0
Late Fry	0	0	0	0	Cabernet Sauvignon	3	2	0	0

z 4, necrotic area >3 mm diameter from wounded spot; 3, necrotic area of 2–3 mm around wounded spot; 2, necrosis spreading to form area on wounded spot; 1, slight necrosis; 0, no necrosis.

**Table 3:** List of oligonucleotide primers used in this study for real-time PCR analysis.

Primer	Orientation	Sequence
CHS	Sense	5′-C(ACT)TATGA(AT)GA(AG)TATCTCTG(CT)-3′
	Antisense	5′-GAGCT(AG)GGAAAAGCCAT(ACT)GT-3′
StSy	Sense	5′-TTGGTATCTGATT(AG)(CG)TGATG-3′
	Antisense	5′-CCAGTA(CT)TC(CT)(CT)GGATGTGTCT(AG)TC(AC)TC-3′
PGIP	Sense	5′-AG(AT)A(AG)(CT)TT(GT)GT(CGT)A(AG)(CT)TGG-3′
	Antisense	5′-TC(AG)(CG)T(GT)AT(GT)AT(CT)TCCAC(AC)AGCAT-3′
CHI	Sense	5′-TCGTGAAAAGAGAAGGGAACTCA-3′
	Antisense	5′-AAAAACGTCTGGAAGCAAAAGC-3′
LIP	Sense	5′-TTGCTCCAGACCTGATTTTTGAT-3′
	Antisense	5′-TGGCACAGTTCAAACATTGCA-3′
Ubiquitin	Sense	5′-TGTCCTCTGTTTACTTGGTGGTAT-3′
	Antisense	5′-CTTCAAGGGTAATGGTCTTCTCAAC-3′

* Ubiquitin was used as an internal control in this experiment—*CHS* (Chalcone Synthase)—*StSy* (Stilbene Synthase)—*PGIP* (Polygalacturonase Inhibiting Protein)—*CHI* (Chitinase)—*LIP* (Lipid Transfer Protein).
